# Antiviral Resistance and the Control of Pandemic Influenza

**DOI:** 10.1371/journal.pmed.0040015

**Published:** 2007-01-23

**Authors:** Marc Lipsitch, Ted Cohen, Megan Murray, Bruce R Levin

**Affiliations:** 1 Department of Epidemiology, Harvard School of Public Health, Boston, Massachusetts, United States of America; 2 Department of Immunology and Infectious Diseases, Harvard School of Public Health, Boston, Massachusetts, United States of America; 3 Division of Social Medicine and Health Inequalities, Harvard Medical School, Boston, Massachusetts, United States of America; 4 Division of Infectious Diseases and General Medicine, Massachusetts General Hospital, Boston, Massachusetts, United States of America; 5 Department of Biology, Emory University, Atlanta, Georgia, United States of America; National Institutes of Health, United States of America

## Abstract

**Background:**

The response to the next influenza pandemic will likely include extensive use of antiviral drugs (mainly oseltamivir), combined with other transmission-reducing measures. Animal and in vitro studies suggest that some strains of influenza may become resistant to oseltamivir while maintaining infectiousness (fitness). Use of antiviral agents on the scale anticipated for the control of pandemic influenza will create an unprecedented selective pressure for the emergence and spread of these strains. Nonetheless, antiviral resistance has received little attention when evaluating these plans.

**Methods and Findings:**

We designed and analyzed a deterministic compartmental model of the transmission of oseltamivir-sensitive and -resistant influenza infections during a pandemic. The model predicts that even if antiviral treatment or prophylaxis leads to the emergence of a transmissible resistant strain in as few as 1 in 50,000 treated persons and 1 in 500,000 prophylaxed persons, widespread use of antivirals may strongly promote the spread of resistant strains at the population level, leading to a prevalence of tens of percent by the end of a pandemic. On the other hand, even in circumstances in which a resistant strain spreads widely, the use of antivirals may significantly delay and/or reduce the total size of the pandemic. If resistant strains carry some fitness cost, then, despite widespread emergence of resistance, antivirals could slow pandemic spread by months or more, and buy time for vaccine development; this delay would be prolonged by nondrug control measures (e.g., social distancing) that reduce transmission, or use of a stockpiled suboptimal vaccine. Surprisingly, the model suggests that such nondrug control measures would increase the proportion of the epidemic caused by resistant strains.

**Conclusions:**

The benefits of antiviral drug use to control an influenza pandemic may be reduced, although not completely offset, by drug resistance in the virus. Therefore, the risk of resistance should be considered in pandemic planning and monitored closely during a pandemic.

## Abstract 2

Emergence of oseltamivir-resistant influenza strains during a pandemic is likely given the heightened selective pressure if the drug is widely used. Marc Lipsitch and colleagues suggest that resistance would reduce but not completely offset the drug's benefits for pandemic control.

## Editors' Summary

**Background.:**

Governments and health authorities worldwide are planning how they would best prepare for and deal with a future influenza pandemic. Seasonal influenza is thought to affect between 5% and 15% of the population worldwide each year. Most people who get influenza recover within a couple of weeks without lasting effects, but a small proportion of patients, mostly young children and elderly people, experience serious complications that can be fatal. An influenza pandemic happens when new variants of the influenza virus emerge against which little immunity exists in the general population. Pandemic influenza strains are transmitted more rapidly than seasonal strains, often sweep across several countries or continents, and make more people ill. There are drugs that can treat and prevent influenza. One of them, oseltamivir (Tamiflu) is an antiviral drug that works by preventing viral particles from being released by infected human cells. Stockpiling large amounts of oseltamivir and related drugs with the intent to treat a large fraction of the population is a key part of pandemic preparedness of many countries. However, it is known that influenza viruses can develop resistance to these drugs.

**Why Was This Study Done?:**

It is not clear how the emergence of oseltamivir-resistant influenza strains would affect the course of any future influenza pandemic. Much research in this area has focused on how likely the new strains are to emerge in the first place, rather than on how they might spread once they had emerged. In the context of an influenza pandemic, antiviral drugs would be used in a large proportion of the population, likely driving the selection and spread of resistant viruses. For this study, the researchers wanted to estimate the likely impact of resistant strains during an influenza pandemic.

**What Did the Researchers Do and Find?:**

These researchers set up a mathematical model (i.e., simulations done on a computer) to mimic the spread of influenza. They then fed a set of assumptions into the computer. These included information about the rate of transmission of influenza from one person to another; what proportion of people would receive antiviral drugs for prophylaxis or treatment; how likely the drugs would be to successfully treat or prevent infection; and in what proportion of people the virus might become resistant to drugs. The modeling led to three main predictions. First, it predicted that widespread use of antiviral drugs such as oseltamivir could quickly lead to the spread of resistant viruses, even if resistant strains emerged only rarely. Second, even with resistant strains circulating, prophylaxis and treatment with oseltamivir would still delay the spread of the pandemic and reduce its total size. Third, nondrug interventions (such as social isolation and school closures) would further reduce the number of cases, but a higher proportion of cases would be caused by resistant strains if these control measures were used.

**What Do These Findings Mean?:**

These findings suggest that, in the event of a future influenza pandemic for which antiviral drugs are used, there is a risk of resistance emerging and resistant strains causing illness in a substantial number of people. This would counteract the benefits of antiviral drugs but not eliminate those benefits entirely. Like all modeling studies, this one relies on realistic assumptions being entered into the model, and it is hard to know closely the model will mimic a real-life situation until the properties of an actual pandemic strain are known. Most studies, including this one, suggest that in the event of a pandemic, antiviral drugs will have an overall beneficial impact on reducing death rates and adverse health outcomes. However, given the sizeable effects of resistance suggested here, its role should be considered in pandemic planning. This includes surveillance that can detect emergence and spread of resistant strains.

**Additional Information.:**

Please access these Web sites via the online version of this summary at http://dx.doi.org/doi:10.1371/journal.pmed.0040015.

- World Health Organization: information on pandemic preparedness
- World Health Organization: fact sheets on influenza
- Information from the UK Health Protection Agency on pandemic influenza
- US government website on both pandemic flu and avian flu (information provided by the US Department of Health and Human Services)

## Introduction

Antiviral drugs, especially the neuraminidase inhibitor (NI) oseltamivir, play a major role in plans to mitigate the next influenza pandemic [1,2]. Current mathematical modeling studies suggest that antivirals alone are unlikely to significantly reduce the spread and impact of a pandemic, in part due to supply constraints [3]. However, these studies also predict that antiviral prophylaxis and treatment may make a significant contribution to pandemic control when combined with other interventions, including social distancing, isolation and quarantine, measures to reduce person-to-person contact such as school closing, and perhaps the use of even a poorly matched but prestockpiled vaccine [4,5]. Moreover, a recently published analysis of interpandemic data (e.g., [6]) suggests that NIs may be more efficacious than assumed in previous models of pandemic control. Although it remains to be seen whether NIs will have appreciable effects on either the clinical course or the transmission of the next pandemic strain [7], many countries plan to rely heavily on them, alongside other measures.

In normal influenza seasons, resistant viruses develop in several percent of individuals who receive oseltamivir treatment [8]. Resistant strains have also arisen in treated hosts infected with avian strains of influenza A/H5N1 [7,9]. Mathematical models designed to consider resistance to the adamantanes (M2 inhibitors) [10] or NIs [11,12] predict that if resistant strains suffer little or no fitness deficit (reductions in transmissibility) relative to sensitive strains, then resistant strains could spread, reducing the effectiveness of antiviral use by a degree that depends mainly on the magnitude of this fitness cost. In such models, the rate of emergence of resistance in individuals who receive treatment or prophylaxis affects the rate at which resistant strains first appear. Once resistant strains have appeared, their ability to spread to many other hosts in the population depends on whether antiviral use provides a sufficiently strong selective force to offset the ”fitness cost” of resistance [13,14].

Mathematical models analyzed to date [11,12] predict that the impact of resistance to NIs on the overall course of the epidemic is largely independent of the probability of de novo emergence of resistance in treated patients, at least for the range of probabilities considered. In contrast, practical discussions of measures to minimize resistance to NIs have focused largely on the need to reduce de novo emergence by appropriate dosing [15,16] and by use of prophylaxis, which is thought to lead to de novo emergence of resistance less often than treatment [17]. One possible reason for this focus on de novo acquisition of resistance is the view that significant transmission of resistant strains is unlikely. Most oseltamivir-resistant viruses appear compromised in replication, infectivity, and/or transmissibility [18]. As frequently noted before, strains bearing such “fitness costs” would be unlikely to spread much beyond the host in whom they first arise [11,12,15]. We believe that these findings, while encouraging, do not settle the issue. In response to a pandemic, developed countries propose to use NIs on up to a quarter or more of the population, potentially upwards of 100 million courses if such use were undertaken just in the United States and the United Kingdom. This would be a dramatic increase over the worldwide use of around 3.2 million courses in the 2001–2002 influenza season [19]. Such increased use of NIs would provide unprecedented opportunities for novel resistant variants to emerge, perhaps including strains with lower fitness costs than previously observed; moreover, widespread use may create selection pressure that could permit spread of a resistant strain despite a significant fitness cost of resistance.

Furthermore, data from interpandemic influenza strains and experience with other pathogens suggest that high fitness costs of resistance are not inevitable. Although two common NI-resistant influenza mutations cause significant fitness deficits in vitro and in vivo, a third (E119V), so far found only in N2 viruses [18], has no detectable fitness cost [20]. In other infections such as HIV and tuberculosis, the frequent presence of fitness costs in resistant mutants [21–23] does not prevent transmission of resistant variants in the population[21,24]. Moreover, compensatory mutations may mitigate any fitness costs incurred by resistant strains [25,26]. Recently published surveillance data show that novel resistance mutations in N1 and N2 neuraminidases continue to appear, including those for which in vitro and in vivo fitness is unknown. Four such novel strains were isolated from individuals with no known history of NI use, suggesting that transmission of resistant strains may have occurred [19]. In summary, it is prudent in pandemic planning to consider the possibility that resistant strains with modest or no fitness cost might emerge at some point during the pandemic, even if most strains observed to date have shown substantial fitness costs [27].

We analyzed a simple mathematical model to examine several key questions about resistance in the setting of a pandemic: (1) To what degree would resistance emerge in the population during a pandemic if de novo emergence of transmissible resistance is very rare? Although the emergence of resistance may occur in a few percent of treated hosts, we wished to consider the possibility that the emergence of strains with limited fitness cost (of greatest interest epidemiologically) might be far lower. (2) What are the relative roles of treatment and prophylaxis in selecting for resistant strains (given that treatment generates de novo resistance at a higher rate than prophylaxis, but prophylaxis blocks transmission of the drug-sensitive strain more effectively than treatment). (3) What is the interaction between antiviral use and the use of other measures to control the spread of influenza? (4) In conditions in which resistance does emerge, does antiviral use reduce the size or delay the peak of the pandemic?

## Methods

Here we describe a homogeneous population model of pandemic influenza and its control by prophylaxis and treatment. The nearly identical age-structured model used in our numerical analysis is described below, and code for the numerical solutions, performed with Berkeley Madonna 8.2 (http://www.berkeleymadonna.com), is provided in Protocol S1 in a form that can be pasted into the free downloadable trial version of Berkeley Madonna.

**Figure 1:**
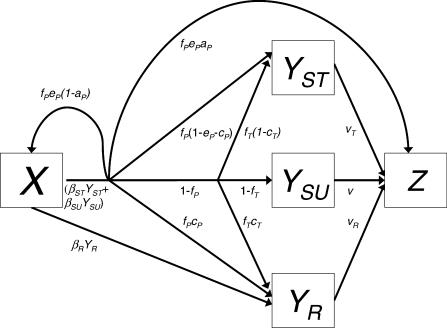
Model Structure Naive *(X)* individuals become infected with sensitive *(S)* or resistant *(R)* strain at rates proportional to prevalence of each strain, *Y_S_* or *Y_R_.* Individuals infected with the sensitive strain may be either treated *(T)* or untreated *(U)*. Individuals are removed *(Z)* by death or recovery. See Methods for corresponding equations. This model was used for analytical calculations, whereas an age-structured version (Methods) was used with parameters shown in Table 1 for numerical solutions.

The basic model (Figure 1) is a standard susceptible-infectious-recovered (SIR) model [28], with compartments representing the number of individuals in each state. All are susceptible at the outset, and births and deaths are neglected on the time scale of interest. The infected/infectious *(Y)* compartment is divided into three categories: infected with sensitive virus and untreated *(Y_SU_),* infected with sensitive virus and treated *(Y_ST_),* or infected with resistant virus *(Y_R_).* In the absence of prophylaxis, transmission occurs at a rate proportional to the number of infectious hosts, with transmission rate constants *β_SU_, β_ST_,* and *β_R_* from untreated and treated hosts infected with sensitive virus, and hosts infected with resistant virus, respectively. Infectiousness is assumed to be exponentially distributed with a mean duration of 1/*v* days.

**Table 1:**
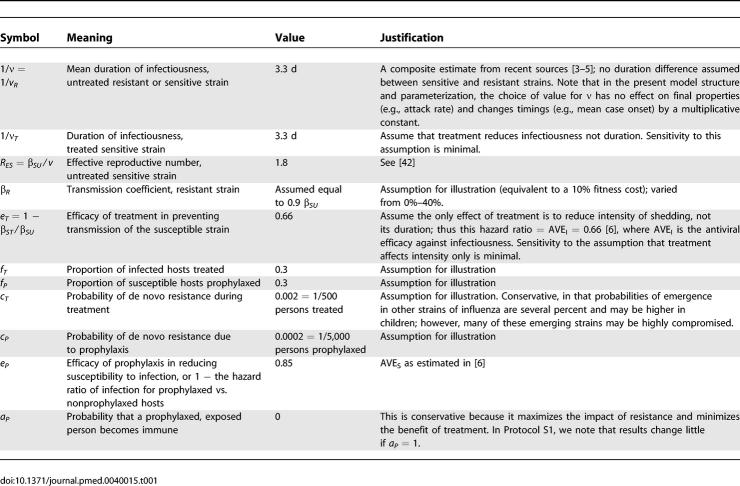
Baseline Parameters Used in the Numerical Examples

A fraction *(f_p_)* of susceptible hosts *(X)* exposed to infection receive prophylaxis. A prophylaxed host who is exposed to infection with the sensitive strain (who would have been infected absent prophylaxis) may have one of three outcomes. With probability *e_p_* (equivalent to AVE_S_ of Longini et al. [3], the reduction in hazard of infection for an individual on prophylaxis), transmission is blocked. Of those blocked infections, a proportion *a_p_* are only partially blocked, so that the prophylaxed individual becomes immune, but does not become sick or infectious to others [17]; such individuals move directly to the recovered *(Z)* category. With probability *c_p_,* the prophylaxis causes de novo resistance, so the individual is infected with drug-resistant virus. In the remaining 1 − *c_p_* − *e_p_* of cases, prophylaxis fails, and the individual is infected with the sensitive virus, but follows a natural history like that of a treated patient [3]. Prophylaxis has no effect on an individual's risk of infection upon exposure to the resistant strain.

Of those persons who become infected but have not been on prophylaxis, a fraction *f_p_* are treated. In a fraction *c_T_* of treated persons infected with the sensitive strain, the strain acquires de novo resistance, turning them (we assume instantaneously) into *Y_R_*. In the remainder, treatment reduces infectiousness to *β_ST_* (versus *β_SU_*) per day, and/or reduces duration of infectiousness from 1/*v* to 1/*v_T_* days. In model runs presented here, the effect is assumed to be only on *β_ST_* ; the code allows the effect to be partitioned arbitrarily between infectiousness and duration, and this distinction has almost no effect on the results. Treatment has no effect on individuals infected with the resistant strain. At the end of the infectious period, individuals enter the removed *(Z)* category; this includes both death and recovery with immunity.

We consider a range of values for the effective reproductive number *R_E_,* specifically, values of *R_E_* = 2.2, 2.0, 1.5, and 1.2. These cover a range of possible basic reproductive numbers for pandemic influenza. Moreover, model runs with the lower values are exactly equivalent to a case in which the basic reproductive number is higher but nonpharmaceutical interventions (e.g., social distancing, mask wearing, quarantine and isolation, etc.) and/or use of a prestockpiled vaccine with partial effectiveness have reduced the transmission of both resistant and sensitive strains. Specifically, consider a virus with *R_0_* = R^*^ in the absence of nondrug interventions. In the presence of interventions that reduce transmission by a proportion *θ,* this virus would transmit identically to a virus with *R_0_* = R^*^(1 − *θ*) in the absence of interventions. Thus, for example, a scenario with *R_E_* = 1.5 could represent either *R_0_* = 1.5 without interventions, or *R_0_* = 2.0 with nondrug interventions reducing transmission 25%, or *R_0_* = 2.2 with nondrug interventions reducing transmission approximately 32%.

### Modeling Low Probabilities of Resistance Emergence

Because we are using a continuous model to evaluate scenarios with a very low probability that resistance will emerge *(c_p_* and *c_T_),* it is important to ensure that we do not generate artifacts by creating a fraction of a resistant case and then allowing that fraction of a case to transmit. To prevent transmission of “nanocases,” we modify the model so that resistant cases accumulate only by de novo emergence until the total number of resistant cases exceeds one; only when there is at least one resistant case can those infections transmit. This is readily accommodated by incorporating an indicator variable

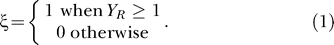



Model equations are:

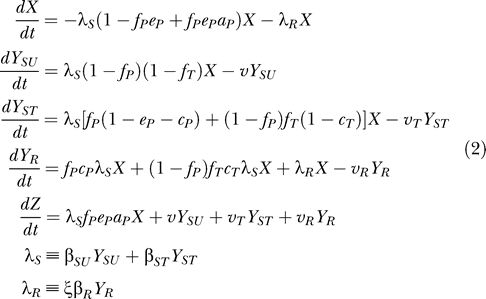



Baseline parameter values used, and their justifications, are given in Table 1. A number of these parameters were varied in sensitivity analyses as shown in the Results and Protocol S1.

### Age-Structured Model

The extent to which individual humans transmit influenza is related to a variety of factors, many of which depend on age. In generating the numerical results, we divided the population into six age groups, *i* = 1 to 6, each with its own initially susceptible population size *X_i_*(0) and force of infection *λ_Si_* and *λ_Ri_*. The restriction on transmission of resistant strains in this model is implemented separately for each age class; hence, once the expected number of resistant infections in class *i* exceeds 1, these infections can be transmitted to individuals in all six age classes. Before that time, resistant infections accumulate in an age class only by de novo acquired resistance and by transmission from other age classes *j* for which *ξ_j_* > 1. The population size distribution and who-acquires-infection-from-whom (WAIFW) matrix were taken from a study of social contact distributions in the Netherlands, with transmission probability per contact calibrated to achieve a desired value of *R_E_* [29]; numerical values are given in Table S1. All numerical results were produced using this model, which differed little from the homogeneous model except that slightly fewer individuals are infected in the entire epidemic (lower attack rate) for a given reproductive number_._

### Treatment and Prophylaxis

**Figure 2:**
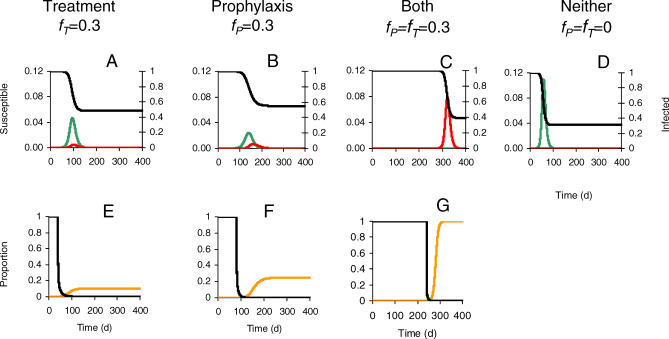
Impact of Treatment, Prophylaxis, Both, or Neither on the Dynamics of Resistant and Sensitive Infections (A and B) Either treatment of 30% of infected hosts (A) or prophylaxis of 30% of contacts (B) reduces transmission of a sensitive strain (green) and leads to selection of a resistant strain (red), with somewhat more resistance due to prophylaxis than treatment, although the risk that resistance will emerge is assumed to be 0.2% for treatment and 0.02% for prophylaxis. Proportions of the entire population infected with either strain are read on the left *y*-axis. Black curves (right *y*-axis) indicate the proportion of the population uninfected. (C and D) Treatment of 30% and prophylaxis of 30% (C) selects most strongly for resistance, because treatment generates resistant strains early on, and these strains are amplified by prophylaxis. (D) shows the impact of neither treatment nor prophylaxis. (E–G) These contrasting effects are apparent in following the cumulative proportion of resistant infections (black lines), which rises earlier with treatment (E), but persists and reaches higher levels with prophylaxis (F). The orange curves show the proportion of all new resistant infections attributable to de novo resistance as opposed to transmission of a resistant strain; the contribution of de-novo resistance is highest early, but rapidly declines. Graph (G) shows the impact of both treatment and prophylaxis.

We initially considered the effects of treatment and prophylaxis separately and together; these results are shown in Figure 2. Thereafter, we considered a combined policy of treating a proportion of *f_T_* of cases and of prophylaxing a proportion of contacts of cases *f_P_*. Combined use of treatment and prophylaxis is likely the most realistic possibility during an actual pandemic [1,4,5]. Because we were interested in the qualitative effects of increased antiviral use, we assumed that these proportions were equal (*f_P_* = *f_T_*) and considered a range of values for these proportions.

We do not explicitly account for the fact that in practice, many of the prophylaxed contacts will be contacts of patients (e.g., household members) who were themselves treated. Sensitivity to this assumption that treatment and prophylaxis are uncorrelated in the population is considered in Section 5 of Protocol S1.

## Results

### Effects of Treatment and Prophylaxis on Resistance

We initially assumed that resistant strains emerge de novo in 0.2% of treated hosts and in 0.02% of exposed contacts under prophylaxis. The figure for treatment is more than 10-fold below those observed in clinical trials [8], but most of the resistant viruses that emerge during treatment appear to have extremely high fitness costs [20]; hence, this can be thought of as a plausible rate of emergence of resistant strains that may under some circumstances be transmissible. Even at these low levels of de novo emergence, treatment or prophylaxis, or both, can produce significant levels of resistance during a pandemic (Figure 2A–2D). Once resistant strains are present, either prophylaxis or treatment favors their spread by impeding the spread of the competing sensitive strain.

Treatment is assumed to generate ten times as many resistant infections de novo for “equivalent” use; nonetheless, for the parameter values we have considered, since prophylaxis is more effective at blocking transmission of the sensitive strain than treatment, prophylaxis creates a greater selective pressure for spread of the resistant strain. Early in the epidemic, when resistance is rare, de novo resistance (and therefore treatment) contributes most to the total resistance in the population (Figure 2E); as resistance becomes more common, transmission becomes more important than de novo emergence, and prophylaxis makes a greater contribution to the spread of resistance (Figure 2F). Use of both prophylaxis and treatment combines these effects, thus efficiently promoting resistance in the population (Figure 2G).

### Effects of Antiviral Use on the Size of the Epidemic

In what follows, we considered the effect of treating a specified fraction of infectious cases while also prophylaxing the same fraction of contacts of infectious cases (see Methods). In the absence of any intervention, the sensitive strain causes an epidemic because its effective reproductive number *R_ES_,* the average number of secondary cases of drug-sensitive infection infected by each drug-sensitive case, exceeds 1. Antiviral use and effective nondrug interventions both reduce transmission of the sensitive strain, and if they reduce it far enough, they can abort the spread of the sensitive strain. Non-drug interventions reduce transmission of both sensitive and resistant strains proportionally, and the resistant strain may have a fitness cost, defined as

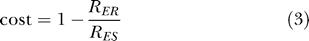
reflecting the physiologic toll of resistance on the virus. The resistant strain can spread if—despite fitness cost and nondrug interventions—its reproductive number *R_ER_* exceeds 1.

**Figure 3:**
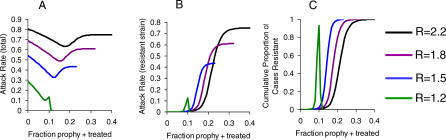
Effect of Varying Effective Reproductive Numbers *(R_E_)* and of Antiviral Use on Total Attack Rate, Attack Rate of the Resistant Strain, and Proportion of All Cases Attributable to the Resistant Strain More-effective nondrug interventions reduce the attack rate while increasing the role of the resistant strain; intermediate levels of antiviral use minimize the attack rate. For combinations of interventions leading to effective reproductive numbers near unity, the epidemic may take an unrealistically long time (three or more years). Prophy, prophylaxed. (A) Total attack rate. (B) Attack rate of the resistant strain. (C) Proportion of all cases attributable to the resistant strain.

**Figure 4:**
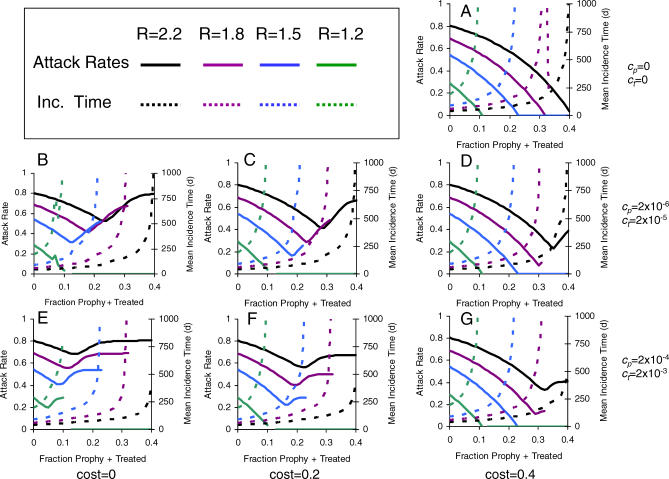
Effect of Varying Effective Reproductive Numbers *(R_E_)* and of Antiviral Use on Total Attack Rate (Solid Curves) and Mean Incidence Time (Dashed Curves) Mean incidence time (“Inc. Time”) is the mean time of infection of all infected persons over the course of the epidemic. Rows represent different frequencies with which resistance emerges during prophylaxis (“Prophy”) (*c_p_* = 0, 2 × 10^−6^, and 2 × 10^−4^) and treatment (*c_t_* = 0, 2 × 10^−5^, and 2 × 10^−3^); columns represent different fitness costs of resistance (0, 20%, and 40%). Since the resistant strain is not present at the start of the simulations, it does not appear when *c_p_* = *c_t_* = 0, hence its fitness cost is irrelevant. Graph (A) represents the case with no resistance. Graphs (B–D) represent the case when *c_p_* = 2 × 10^−6^ and *c_t_* = 2 × 10^−5^ at a fitness cost of 0, 0.2, and 0.4, respectively. Graphs (E–G) represent the case when *c_p_* = 2 × 10^−4^ and *c_t_* = 2 × 10^−3^ at a fitness cost of 0, 0.2, and 0.4, respectively.

Antiviral use retards the spread of the sensitive strain, reducing the total number of individuals infected during the epidemic (attack rate) (Figure 3A). Use of treatment and prophylaxis in such a large epidemic inevitably generates resistant strains. However, with modest amounts of antiviral use, the epidemic is largely due to the sensitive strain, which is more common at the start of the epidemic than the resistant strain. Spread of the sensitive strain depletes the pool of susceptible hosts before the resistant strain has time to spread widely. Thus, moderate levels of treatment and prophylaxis reduce the attack rate by impeding spread of the sensitive strain without promoting extensive spread of the resistant strain (Figure 3B). At higher levels of use, however, *R_ES_* is further reduced, allowing greater spread of the resistant strain so that it is able to infect a large proportion of the population before the sensitive strain does so. Thus, perversely, at higher levels of antiviral use, the attack rate increases again. For the levels of antiviral use considered so far, intermediate levels of use are the most effective in reducing the attack rate; low levels have minimal impact, whereas very high levels promote the spread of the resistant strain too quickly. The exact value of this “optimal” level of use depends on the values of such parameters as the fitness cost of resistance, the transmissibility of both viruses in the presence of nondrug interventions *(R_ES_* and *R_ER_),* and whether prophylaxis allows individuals to become immune (Figures 3 and 4). The mechanism for this phenomenon is examined in the Discussion and in Protocol S1.

As one considers still higher levels of antiviral use, the attack rate drops down to nearly 0 in the model. If antiviral use is high enough to bring the effective reproductive number of the sensitive strain to near or below 1, then it is possible that no resistant strain will be created, because so few sensitive cases will be treated that the expected number of resistant cases emerging de novo will be less than 1. Thus, at very high levels of antiviral use, the attack rate is close to 0, since the sensitive strain's spread is blocked almost completely, and the resistant strain does not emerge. For this to occur for the parameter values used in Figure 3B, there must be fewer than approximately 500 (= 1/*c_T_*) sensitive cases treated, or (at 40% treatment) fewer than 1,250 sensitive cases total, to prevent the emergence of a resistant strain. Thus, this best of all possible outcomes, although possible in theory, requires that high levels of antiviral use and other interventions be implemented very early to essentially stop the spread of the sensitive strain. It also requires that no resistant strains be imported from elsewhere. Such effective interventions are difficult in any one location, and almost inconceivable worldwide, hence, we do not expect that this theoretically possible outcome will come to pass.

### Effects of Nondrug Interventions

The four curves in each graph of Figure 3 can represent either different basic reproductive numbers for the sensitive strain (reflecting viral properties and population contact patterns) or, equivalently, varying levels of nondrug interventions (lower *R_E_* corresponding to more-effective nondrug interventions). We describe results in terms of the latter. Regardless of antiviral use or resistance, more-effective nondrug interventions, of course, lead to lower overall attack rates (Figure 3A), whereas greater antiviral use leads to higher attack rates with the resistant strain (Figure 3B) and higher proportions of all cases attributable to the resistant strain (Figure 3C).

In the model, antiviral use selects more effectively for resistance when the reproductive number of both strains is lower, either intrinsically or due to the presence of more-effective nondrug interventions (Figure 3B), though the total attack rate (with sensitive and resistant strains) is lower when transmission is reduced (Figure 3A). Viewed another way, resistant cases are a greater proportion of the total in the presence of nondrug interventions than without them (Figure 3C). The reason for this finding is considered further in the Discussion and in Protocol S1.

### Effects of Resistance on Epidemic Size

Significant spread of a resistant strain partially compromises the benefits of antiviral use (Figure 3A). Even with extensive spread of the resistant strain, the attack rate is less than that which obtains in the absence of antiviral use (Figure 3A). This reflects the assumed lower transmissibility of the resistant strain (fitness cost), as well as the fact that successive epidemics of the sensitive and resistant strains together infect fewer people than the sensitive strain alone would have if unchecked (Protocol S1).

By slowing the spread of the sensitive strain, antiviral use delays the peak of the epidemic (Figure 2). Although it also promotes the ascent of resistance, resistant strains (if initially rare) will take time to infect enough hosts to make a noticeable contribution to the epidemic (Figure 4). Any fitness cost of resistance further slows the resistant epidemic. Taken together, these effects can substantially increase the time until the average infection occurs, especially if combined with effective nondrug interventions (Figure 4). Such delays may provide time for vaccine production, while spreading impacts on society and the health care system over a longer time and reducing peak loads. If pandemic influenza transmission is seasonal, such delays may be extended by declining transmission in the summer months; however, resistant strains might persist in the population (at a similar or different frequency) [30] to reappear in the next season.

### Dependence of Outcomes on Fitness Cost and Intensity of Control

Overall, the benefit of antiviral use in the model depends strongly on the fitness of the resistant strain and on the intensity of control measures. Prior studies [3–5,11] and our model predict that in the absence of resistance, antiviral use that blocks a substantial fraction of transmission, combined with other transmission-blocking interventions, can slow or even stop transmission of an influenza epidemic (Figure 4A). These benefits are partially compromised by the emergence of resistance (Figure 4B–4G), and the degree of compromise depends strongly on several factors. Most apparent is the fitness cost of resistance. If this cost is 20% or greater (Figure 4C, 4D, 4F, and 4G), then even if resistant strains constitute nearly the whole epidemic due to heavy antiviral use, the attack rate may be reduced by tens of percent of the entire population, and the epidemic can be delayed by a few or even many months. However, if there is no fitness cost of resistance, and if the transmissibility of both resistant and sensitive strains is high (black curves), the reduction in attack rate is 10% or less, and the delay is no more than a few months (Figure 4B and 4E).

Successful nondrug interventions, which themselves reduce the attack rate, considerably amplify the benefits of antiviral use, even in the presence of resistance (Figure 4, blue and green curves compared to purple and black curves). This occurs for two reasons. Highly effective nondrug interventions, combined with high levels of antiviral use, might in principle abort the epidemic of the sensitive strain before a resistant strain is generated; we have discussed above why this scenario is probably unlikely in practice. More realistically, the combination of nondrug interventions and fitness cost substantially reduce the reproductive number of the resistant strain. If both strains have reproductive numbers below 1, the attack rate approaches 0 since neither strain can spread effectively (blue and green curves, *f_T_* = *f_P_* > 0.1 to 0.2).

Our sensitivity analyses suggest that even when resistant strains emerge at seemingly low frequencies (0.002% for treatment and 0.0002% for prophylaxis), resistance may significantly erode the benefits of large-scale antiviral use, especially if the pandemic strain is highly transmissible (Figure 4B and 4C). Resistant strains emerging in hosts who are treated or prophylaxed will probably be genetically heterogeneous and vary in their fitness costs [20]. Over time, the lower-cost strains will play a disproportionate part in spreading to other hosts [31], and their spread is of the greatest concern. We have considered emergence probabilities well below those observed, to represent the possibility that, for example, resistance emerges in 4% of treated hosts [7,8,16], of which 0.05% are a strain with modest fitness cost.

## Discussion

In the absence of a resistant strain, our model, like others before it, predicts that use of an effective antiviral drug combined with other effective measures to reduce transmission could reduce the size of a pandemic and delay its onset, buying valuable time for vaccine production. If all resistant strains have very high fitness costs (e.g., 40% or more), or if the spread of the sensitive strain could be aborted within the first few thousand cases, our model suggests that the spread of resistant strains could be negligible. Such scenarios, although possible, seem unlikely. To date, most but not all strains resistant to the NIs seem to be handicapped in their ability to transmit from host to host [20], but the possibility that higher-fitness strains (with costs substantially less than 40%) might emerge must be considered, given the unprecedented levels of NI use anticipated during a pandemic. Indeed, we have considered very low probabilities of emergence (as low as 1/50,000 treated cases and 1/500,000 prophylaxed cases) and find that, if strains with little or no fitness cost emerge at these rates, resistance could spread widely in a pandemic (Figure 4B and 4C). Although effective measures to control an emerging pandemic may be possible for a closed community, it seems highly implausible that these effective measures could be implemented at a global level. As a result, if resistant strains capable of transmission emerge in some communities, introduction of these strains would be expected even into communities that have effectively controlled the epidemic from the start and averted the appearance of “home-grown” transmissible resistant strains.

Taken together, these arguments suggest it is important to consider the possibility that resistant strains with modest fitness costs (say 20% or less) can emerge at even extremely low frequencies de novo in hosts receiving antiviral drugs. The model suggests that, if this is the case, resistant strains may well may make a significant contribution to a pandemic. Although this conclusion is pessimistic, it is balanced to some degree by the model's prediction that, even if resistant strains do spread widely, the use of antivirals will likely reduce the magnitude and delay the peak of a pandemic. The extent of this delay depends on the exact magnitude of the fitness cost (higher cost equals more delay), the amount of antiviral use (more use equals more delay), and the effectiveness of other, nondrug interventions (better interventions/less transmissibility equals more delay).

One of the more surprising results obtained here is that (except for the unlikely case in which antiviral use can stop the epidemic before a resistant strain appears) the benefit of antiviral use in reducing the attack rate is greatest for intermediate levels of antiviral use. The reason for this prediction can be seen by imagining two extremes: Case A, in which no antiviral use occurs, so the epidemic simply unfolds without hindrance from antivirals but without resistance, and Case B, in which every individual is treated and prophylaxed throughout the epidemic. Assuming that a resistant strain capable of transmission emerges in Case B, the epidemic will essentially be all resistant, but again (because the antivirals are ineffective against the resistant strain), the resistant epidemic will pass unhindered by treatment. If we further assume that the fitness cost of resistance is 0 or minimal, then Case A and Case B will be almost identical in terms of attack rate, since each will be an epidemic of an essentially identical virus (from the point of view of transmission). Now consider Case C, with an intermediate level of antiviral use, which is not enough to stop transmission of the sensitive strain. Then there will be an epidemic of the sensitive strain, smaller than in Case A because antivirals slow its spread, but followed (at some interval, longer if emergence of resistance is rare) by an epidemic of the resistant strain. Because the resistant strain will be spreading in a population that is already partially immune (thanks to prior exposure to the sensitive strain), it will spread less successfully than in Case B, and in particular will “overshoot” less in the total number of people it infects. Here, overshoot refers to the standard property of epidemics in closed populations of peaking when they have reduced the number of susceptibles to a level that can no longer maintain growth of the epidemic (in simple models, to 1/*R_0_*), but continue to infect more as the epidemic declines, so that the proportion of the population that escapes infection by the end of the epidemic is less than 1/*R_0_*. The precursor epidemic of the sensitive strain reduces this overshoot and thereby results in a smaller attack rate. This phenomenon is explained more generally and more quantitatively in Protocol S1.

Another surprising result obtained here is that resistance reaches higher levels in the epidemic when more-successful nondrug interventions are in place, or equivalently when the baseline transmissibility of the infection is lower. This phenomenon, considered more quantitatively in Protocol S1, can be understood intuitively as follows. When transmission is highly efficient (due to a high baseline reproductive number or ineffective nondrug interventions), the epidemic happens so fast that resistance does not have time to reach very high levels; if, instead, the epidemic is slower, due to a less transmissible virus or better nondrug interventions, resistance has time to take off before the epidemic is over. More quantitatively, the prevalence of resistance in the population increases approximately by a factor proportional to *R_R_* − *R_S_* in each epidemic “generation” (the time from infection of one person to infection of his contact, which is on average 1/*ν*). When more-effective nondrug interventions are in place, this increase is somewhat smaller per generation (since *R_R_* and *R_S_* are reduced), but that reduction is more than offset by an increase in the number of generations of transmission in the epidemic (note that in Figure 4, the mean time to infection, a measure of the number of generations in the epidemic, can be two or more times as long when the reproductive number is reduced by 25% from 2.0 to 1.5). As an extreme limiting case, imagine an infection that had a basic reproductive number comparable to the population size, so that nearly the entire population was infected by the first index case. Even if everyone were prophylaxed and the index case treated, resistance would occur only in those who acquired it de novo; the epidemic would be over after one (or slightly more) generations, before resistance had time to increase much above its de novo emergence rate through repeated transmission over several epidemic generations.

The simple model used here enables evaluation in a general way of the potential impact of resistance on an influenza control program, to assess the relative contributions of different factors (parameters) contributing to the epidemic. It uses a simplified, exponentially distributed natural history; however, since most of the conclusions (except for those about the magnitude of delays) are about the final state of the population, and all terms in the equations can be nondimensionalized by dividing by the generation time, this simplification does not have a major effect on outcomes. This model does not account for saturation of contacts due to transmission within families and other small groups, and therefore predicts higher attack rates than more complex, agent-based models [4,5] for a given value of *R_0_*. Given the simplifications inherent in any model and the large uncertainties about the properties of a potential pandemic strain and its resistant variants, we emphasize the qualitative predictions of the model—supported by analytical reasoning in Protocol S1—rather than the exact quantitative predictions. Important predictions that we believe to be robust to model structure are that (1) antiviral use will favor the spread of resistance even if such use rarely generates de novo resistant strains; (2) despite the spread of resistance, prophylaxis and treatment can both delay and reduce the size of the epidemic; (3) nondrug interventions (if effective) and antiviral use—which will likely be used together in the response to a pandemic [1,4,5]—generally have synergistic benefits, despite the fact that nondrug interventions may promote resistance; and (4) relatively minor differences in fitness cost may make large differences in outcomes, even when emergence probabilities are low (Figure 4). These results extend those of previous models, which showed (like our model) that the fitness cost of resistance strongly influences the ability of resistant strains to spread during an epidemic [10–12].

It may be possible to reduce the risk of amplifying resistant viral subpopulations within a treated host [7,8,15,32] by adequate dosing and greater emphasis on prophylaxis than treatment [16]. However, our results suggest that once resistant strains are present in the population, as they likely will be [7,33], heavy use of antivirals even for prophylaxis will promote their spread by inhibiting the drug-sensitive strain that competes with them. Thus, even a highly effective antiviral regimen with little or no risk of producing de novo antiviral resistance will, when used population-wide, promote the spread of resistant strains. Similar phenomena are observed in models of Mycobacterium tuberculosis [34], Streptococcus pneumoniae [35], and hospital-acquired infections [36], and are of concern when considering preventive treatment of tuberculosis [37] and malaria [38].

The conclusions described here are specifically relevant to NIs; prospects for the use of M2 inhibitors (adamantanes) in a pandemic are considered far less promising. Adamantane resistance is very widespread in some populations of H5N1, though rare in others [39]. The resistance profile of a strain causing the next pandemic is, of course, unknown. However, even if the pandemic were initially susceptible to adamantanes, widespread use these agents would likely select for resistance extremely rapidly [10], because rates of de novo emergence are considerably higher [8] for these agents than for NIs, whereas fitness costs for adamantane resistance are unmeasurable [40].

Although adamantane monotherapy is unlikely to be effective, combination chemotherapy with both NIs and adamantanes might have some benefits against a pandemic strain initially susceptible to both classes, just as combination therapy has been useful in other infections [41]. First, the rate of de novo emergence of resistance within hosts might be reduced. Second, and perhaps more importantly, the spread of mutant strains resistant to only one of the drugs might be curtailed in the population, because treatment and prophylaxis with the drug combination might reduce susceptibility and/or infectiousness even if only one of the drugs is effective. Finally, it is at least theoretically possible that dually resistant strains would suffer a greater fitness cost than singly NI-resistant strains, although this benefit may be negligible given the high fitness of adamantane-resistant strains.

Optimism about the benefits of antivirals in an influenza pandemic should be tempered by the knowledge that transmissible, pathogenic resistant strains are a real possibility and could reduce the benefits of antiviral use in pandemic control. Successful implementation of nondrug interventions to control resistance will, in most circumstances, amplify the benefits of antiviral use in controlling the pandemic, although such interventions may increase the proportion of resistant cases. Because the impact of resistance is relatively insensitive to the rate at which resistant strains emerge de novo in antiviral recipients, efforts to control influenza transmission overall may be of greater benefit than efforts to reduce the de novo rate of emergence of resistance. Despite these caveats, we do not believe that concerns about resistance should preclude the widespread deployment of antivirals as part of the response to a pandemic. If these drugs, used prophylactically or for treatment, are effective in reducing transmission of the next pandemic strain, they should provide benefits by reducing the number of infected patients and delaying transmission, even if resistant strains ultimately become widespread.

## Supporting Information

Protocol S1 Supplementary Methods, Analytic Results, and Simulation Code (271 KB PDF)

Table S1 Population Sizes and Transmission Coefficients for the Age-Structured Model The daily transmission rate constant in row *i,* column *j,* indicates that an infected individual in age group *j* has a daily probability of transmitting infection *β_SUij_* to each of the susceptible individuals in group *i*. Note that table entries are inflated by a factor of 10^9^ for readability. This matrix is calibrated for *R_0_* = 2.
(30 KB DOC)

## Abbreviations

NI: neuraminidase inhibitor
