# Supplementary material for: Antiviral Resistance and the Control of Pandemic Influenza
Source: PLoS Med. 2007 Jan 23;4(1):e15. doi: 10.1371/journal.pmed.0040015 (PMC1779817; doi:10.1371/journal.pmed.0040015)
Supplement: Supplementary file 1 [file pmed.0040015.sd001.pdf]

PROTOCOL S1

1. AGE-STRUCTURED MODEL EQUATIONS.

The full age-structured model recapitulates the single-group model shown in the main text, but with six copies of each of the model equations, with the force of infection indexed by *i*=1 to 6 for the 6 age groups. Starting susceptible population sizes are those from the Netherlands in ref. [1], while the force of infection for age group *i* is given by:

$$\lambda_{Si} \equiv \sum_{j=1}^{6} (\beta_{SUij} Y_{SUj} + \beta_{STij} Y_{STj})$$
$$\lambda_{Ri} \equiv \sum_{j=1}^{6} \xi_j \beta_{Rij} Y_{Rj} .$$

These population sizes and values of β_{SUij} are given in Supplementary Table 1. The values of the β_{SUij} are calculated from the contact matrix defined in [1], scaled linearly to obtain a maximum eigenvalue of R_{ij} equal to the desired value (2 in Supplementary Table 1). No aging of the population is considered in the brief interval simulated. Note that the ξ_j term is indexed by *j*, so that as soon as at least one resistant case has (probabilistically) accumulated in an age stratum, it can transmit to all other age strata. Thus in the age-structured model, resistant cases accumulate by *de novo* emergence and by transmission from other age strata in which at least a single case has accumulated.

2. QUASI-STEADY STATE APPROXIMATION.
To facilitate analytic calculations, we make the following quasi-steady state approximation of the full system of equations.

Note that $q \equiv 1 - \frac{(1-f_P)(1-f_T)}{(1-f_P)(1-f_T c_T) + f_P(1-e_P - c_P)}$ is the proportion of incident cases infected with the sensitive strain who are treated (this excludes those in whom resistance emerges, since that event is assumed to occur at the start of infection). Since transmission is from prevalent cases, we are interested in the proportion of prevalent sensitive cases who are treated; this is different from the incidence proportion because duration is different under treatment. Define this prevalence proportion as ϕ_T. Assume that ϕ_T takes on its quasi-steady-state value, $\phi_T = \frac{qv}{qv + (1-q)v_T}$. This QSS assumption allows us to simplify the system to include only one equation for persons infected with the sensitive strain, in which infectiousness and clearance rates are weighted averages of those for treated and untreated persons:

$$
\begin{aligned}
\frac{dX}{dt} &= -\lambda_S(1 - f_P e_P + f_P e_P a_P)X - \lambda_R X \\
\frac{dY_S}{dt} &= \lambda_S[(1-f_P)(1-f_T c_T) + f_P(1-e_P - c_P)]X - v_S Y_S \\
\frac{dY_R}{dt} &= f_P c_P \lambda_S X + (1-f_P) f_T c_T \lambda_S X + \lambda_R X - v_R Y_R \\
\frac{dZ}{dt} &= \lambda_S f_P e_P a_P X + vY_{SU} + v_T Y_{ST} + v_R Y_R \qquad (2) \\
\lambda_S &\equiv [\beta_{SU}(1-\phi_T) + \beta_{ST}\phi_T]Y_S \\
\lambda_R &\equiv \beta_R Y_R \\
v_S &\equiv v(1-\phi_T) + v_T \phi_T
\end{aligned}
$$

Finally, we rewrite the system once more, now only redefining parameter combinations for readability:

$$\begin{aligned}
\frac{dX}{dt} &= -(b_S + b_{SR})Y_S X - b_R Y_R X - \lambda_S f_P e_P a_P X \\
\frac{dY_S}{dt} &= b_S Y_S X - v_S Y_S \\
\frac{dY_R}{dt} &= b_{SR} Y_S X + b_R Y_R X - v_R Y_R \\
\frac{dZ}{dt} &= \lambda_S f_P e_P a_P X + v Y_{SU} + v_T Y_{ST} + v_R Y_R \\
v_S &\equiv v(1-\phi_T) + v_T \phi_T \\
b_S &\equiv [(1-f_P)(1-f_T c_T) + f_P(1-e_P-c_P)][\beta_{SU}(1-\phi_T)+\beta_{ST}\phi_T] \\
b_{SR} &\equiv [f_P c_P + (1-f_P) f_T c_T][\beta_{SU}(1-\phi_T)+\beta_{ST}\phi_T] \\
b_R &\equiv \beta_R
\end{aligned} \tag{3}$$

Of interest will be the change in the ratio of resistant to sensitive strains in the population, $\rho \equiv \frac{Y_R}{Y_S}$. Note that, from system (3) and the quotient rule,

$$\frac{d\rho}{dt} = [(b_R - b_S)X - (v_R - v_S)]\rho + b_{SR}X \ . \tag{4}$$

In time steps of the duration of infectiousness for the resistant strain ($1/v_R$) we have:

$$\frac{1}{v_R}\frac{d\rho}{dt} = [(R_R - R_S K)X + K - 1]\rho + R_{SR}XK \tag{5}$$

where $R_R \equiv b_R/v_R$ is the effective reproductive number for the resistant strain; $R_S \equiv b_S/v_S$ is the effective reproductive number for the sensitive strain given the current level of treatment and prophylaxis; $R_{SR} \equiv b_{SR}/v_S$ is the effective “reproductive number” for resistant cases created by sensitive cases given current levels of treatment and prophylaxis; and $K \equiv v_S / v_R$ is the ratio of the mean duration of resistant to the mean duration of sensitive infections, given the level of treatment and prophylaxis in the population. As we have set up the model, treatment reduces R_S and increases K, while prophylaxis reduces R_S. This quasi-steady state approximation is exact when the effect of treatment is on infectiousness only ($\beta_{ST} < \beta_{SU}, v_T = v$) and approximate when the effect is on duration as well ($v_T > v$). The approximation works well through the peak of the epidemic but becomes less good in the declining phases.

NOTE: $\rho \equiv \frac{Y_R}{Y_S}$ is convenient for analytic tractability because of the logistic structure of the equations of this system. The instantaneous proportion of resistant infections can be recovered, of course, as $\frac{Y_R}{Y_R + Y_S} = \frac{\rho}{1+\rho}$. While ρ is the simplest quantity to study dynamically, we have in the text and Figs 2 and S1 considered a different variable, the cumulative proportion of all infections that are resistant, given by

$$\frac{\int_0^\infty v_R Y_R(t)dt}{\int_0^\infty [v_R Y_R(t) + v_T Y_{ST}(t) + vY_{SU}(t)]dt}$$

(here the weighting of cases by clearance rate converts integrated prevalence to total incidence). This is more informative for considering the whole epidemic (as opposed to initial rates of increase) because the proportion resistant is of less interest when there is only one case than when there are many cases, and the cumulative incidence measure here takes that differenc into account in an appropriate way.

3. ADDITIONAL RESULTS

3a. *Assumptions about the effect of prophylaxis on immunity have modest effects on outcomes.* We have shown results under the assumption that individuals exposed to infection and successfully protected by prophylaxis do not make an effective immune response, hence remain in the susceptible (*X*) rather than the removed (Z) category ($a_p = 0$). As expected, other extreme assumption, that all such individuals become immune ($a_p = 1$), modestly reduces the attack rate for a given level of prophylaxis (results not shown).

3b. Parameterization of antiviral treatment effect on infectiousness has little effect on results. In the main text we make the assumption that the duration of infectiousness is the same for treated and untreated hosts infected with drug-sensitive virus, but the intensity is multiplied by $1-e_T$. More generally, for a given efficacy of treatment in reducing transmission, we could assume that the mean duration of infectiousness is multiplied by $(1-e_T)^{\gamma}$ and the intensity of transmission is multiplied by $(1-e_T)^{1-\gamma}$. Changing γ between 0 and 1 had minimal impact on the results (not shown).

4. ANALYTIC RESULTS

4a. When resistance is rare, de novo emergence of resistance is more important than transmission of resistance; however, as resistance becomes common, de novo becomes less important than transmission. Intuitively, this simply means that when resistance is rare, each additional case of resistance generated by de novo emergence is important, but once it is common, secondary cases of resistance become more common and come to dominate the effect of de novo emergence. This qualitative result is clear from equation (5), in which there are two terms contributing to the rise in the odds of resistance in the population. In the absence or near-absence of resistance ($\rho \to 0$), the last term, which is always positive and describes the *de novo* emergence of resistance due to treatment and/or prophylaxis, dominates equation (5). When resistance becomes more common ($\rho > 0$), the first term may dominate. The first term describes the differential transmission, $(R_R - R_S K)X$, and survival, $K-1$, of the resistant vs. the sensitive strain. The first term is typically negative in the absence of any antiviral use, reflecting the fitness cost of resistance in the form of lower transmissibility ($\beta_R < \beta_{SU}$) and/or faster clearance ($v_R > v_{SU}$) of the resistant strain. However, the first term increases with treatment, which reduces the duration and/or transmissibility of the sensitive strain, while leaving the resistant strain unaffected. Thus, if there is enough treatment and/or prophylaxis to offset the fitness cost of resistance, then the first term will become positive and (if ρ is big enough) dominate the second term.

4b. When resistance is rare, treatment will contribute more than prophylaxis to the growth of resistance in the population. Given our assumption that prophylaxis reduces transmission more than treatment, prophylaxis contributes more than treatment when resistance is sufficiently common. We can compare the marginal contribution of treatment (f_T) vs. that of prophylaxis (f_P) to the rate at which resistance grows, $\frac{d\rho}{dt}$, assuming (as in the text) that treatment reduces infectiousness but not duration (similar calculations, but more involved, can be made when both are affected). We calculate the sensitivities of $\dot{\rho} = \frac{d\rho}{dt}$ to treatment and to prophylaxis:

$$\begin{aligned}\sigma_P &\equiv \left.\frac{d\dot{\rho}}{df_P}\right|_{f_P=f_T=0} = \rho[\beta_{ST}(1-c_P-e_P)-\beta_{SU}]+\beta_{SU}c_P \\ \sigma_T &\equiv \left.\frac{d\dot{\rho}}{df_T}\right|_{f_P=f_T=0} = \rho[\beta_{ST}(1-c_T)-\beta_{SU}]+\beta_{SU}c_T\end{aligned} \qquad (6)$$

Clearly, when resistance is rare (ρ small), the sensitivity to treatment is greater, since by assumption treatment leads to more acquired resistance than prophylaxis. When resistance is common (ρ large), prophylaxis contributes more under the biologically reasonable assumption that the reduction in transmission by prophylaxis is greater than the frequency of emergence of resistance under treatment ($e_P > c_T$).

To clarify the algebra, this calculation has been made for the first unit of prophylaxis or treatment (derivative evaluated at $f_P = f_T = 0$). This assumption is required only for the last term of each of the sensitivities (which does not contain ρ); more generally, it is easy to show that for sufficiently large ρ, $d\rho / dt$ is more sensitive to a unit increase in prophylaxis than treatment for any equal frequencies of treatment and prophylaxis (evaluating the sensitivities at $f_P = f_T = f$)

NOTE: This comparison may be slightly misleading, since we are comparing the fraction of *infectious* hosts treated vs. the fraction of *susceptible* hosts prophylaxed. This is not the same amount of drug use, since there will almost always be more susceptible hosts than infectious ones – requiring more drug for prophylaxis than treatment of the same "fraction."

4c. Explanation of the finding that reducing transmission by non-drug interventions increases the fraction of resistant cases in the epidemic as a whole. We note in the Main Text and Fig. 3 that control of transmission by social distancing, vaccination, etc. will slow the epidemic and reduce overall attack rates, but will increase the prevalence of resistance. In the Main Text we state that this can be understood as a "race" between the drug-sensitive and drug-resistant epidemics, in which slowing transmission allows more time for the resistant epidemic to "catch up."

More formally, this result can be clearly understood in terms of Equation (5) above. Assuming (as we have throughout) that antiviral treatment reduces infectiousness throughout the duration of infection, but does not reduce the duration of infectiousness (*K*=1), Equation (5) shows that the exponential growth phase of the prevalence of resistance occurs with a time scale of $\tau_{RES} \sim \frac{1}{R_R - R_S}\frac{1}{v}$, where $\frac{1}{v}$ is the duration of infectiousness or the "generation time" of the epidemic. The growth of the epidemic of the sensitive strain occurs on a time scale of $\tau_{EPID} \sim \frac{1}{R_S - 1}\frac{1}{v}$, which comes directly from the expression for the rate of exponential growth of an epidemic in a standard SIR model: $r = (R_0 - 1)v$ [2]. Reducing transmission of both strains by a factor $1-\theta$ (say by social distancing) increases τ_{RES} by a factor $\frac{1}{1-\theta}$ but increases τ_{EPID} by a

factor $\frac{R_S - 1}{R_S(1-\theta)-1} > \frac{1}{1-\theta}$. Thus, the time scale of the epidemic slows by more than the time scale on which resistance increases, giving resistance more time to "catch up" before the epidemic has passed through the population.

4d. Explanation of the intermediate "optimum" in control of the epidemic.
Figures 3 and 4 in the main text show that, when resistance is able to spread, the total attack rate can be minimized by an intermediate amount of antiviral use. If less than this amount is used, the sensitive strain infects more people, while if more than this amount is used, the resistant strain spreads quickly and essentially causes the whole epidemic.

The basic mechanism behind this finding is that epidemics "overshoot." In a basic SIR model, an epidemic can "take off" only if the proportion of the population susceptible exceeds $1/R_0$. However, once the epidemic takes off, it "overshoots," continuing to spread even after the proportion susceptible goes below this number. Moreover, the larger the starting susceptible population, the larger the overshoot, and the fewer susceptibles are left at the end of the epidemic, for a given R_0.

Our finding is that the proportion of the population left susceptible after an uncontrolled epidemic of the sensitive strain with a given R_0 (call it R_{0S}) is smaller than the proportion left susceptible after a partially controlled epidemic with that same strain (whose reproductive number is reduced by prophylaxis to $R_{PS} < R_{0S}$), followed (possibly) by an epidemic among the remaining susceptibles with a resistant strain, which has a basic reproductive number of $R_{0R} \le R_{0S}$.

This may happen in one of two ways. First, the partially controlled epidemic may be large enough that it leaves too few susceptibles for the resistant strain to spread at all. If so, it will nonetheless have left more susceptibles than an uncontrolled susceptible strain, since it was partially controlled, thus providing a net benefit.

Second, the partially controlled epidemic may have left enough susceptibles for the resistant strain to spread, specifically it may have left at least a proportion $1/R_{0R}$ of the population susceptible. Let us consider the worst case, of no fitness cost, $R_{0R} = R_{0S}$. Then to show that the total size of the controlled sensitive epidemic plus the resistant epidemic is smaller than the size of the sensitive epidemic would have been without control, it is sufficient to show that the proportion left susceptible at the end of an epidemic, for a given R_0, is a decreasing function of the proportion susceptible at the start of the epidemic: in particular, more susceptibles will be left after the resistant strain spreads in a

partially immune population than if a sensitive strain with the identical R_0 had spread in a fully immune population. This can be done by a simple calculation recapitulating the standard final size calculation for an SIR epidemic[2,3].

Consider the SIR epidemic without births or deaths:

$$dS/dt = -\beta SI$$
$$dI/dt = \beta SI - vI \; .$$
$$dR/dt = vI$$

Now divide the first by the second equation and get

$$\frac{dI}{dS} = (dI/dt)/(dS/dt) = -1 + \frac{v}{\beta S}$$
$$dI = \left(-1 + \frac{v}{\beta S}\right) dS$$
$$\int_0^\infty dI = \int_0^\infty \left(-1 + \frac{v}{\beta S}\right) dS$$
$$0 = I(\infty) - I(0) = S(0) - S(\infty) + \frac{v}{\beta}(\ln S(\infty) - \ln S(0)).$$

We wish to show that the proportion susceptible at time ∞ increases as the proportion susceptible at time 0 decreases, for a given transmissibility, i.e. $\frac{dS(\infty)}{dS(0)} < 0$. This is easily shown by differentiating the preceding equation with respect to $S(0)$:

$$\frac{d}{dS(0)}\{S(0) - S(\infty) + \frac{v}{\beta}(\ln S(\infty) - \ln S(0))\} = 0$$
$$1 - \frac{dS(\infty)}{dS(0)} = \frac{v}{\beta}\left(\frac{1}{S(\infty)}\frac{dS(\infty)}{dS(0)} - \frac{1}{S(0)}\right)$$
$$\frac{dS(\infty)}{dS(0)} = \frac{\frac{\beta}{v} - \frac{1}{S(0)}}{\frac{\beta}{v} - \frac{1}{S(\infty)}} = \frac{+}{-} < 0$$

QED.

We note that this argument relies on many of the same considerations as a similar argument recently made by Handel et al. (Andreas Handel, Ira Longini and Rustom Antia, manuscript submitted to *P Roy Soc Lond B*, 2006)

5. STRUCTURAL SENSITIVITY. Correlation between treatment of cases and prophylaxis of their contacts.

In the main analysis, we made the simplifying assumption that the probability of prophylaxing a particular contact was the same (f_P) regardless of whether the index case of that contact was treated or not. This assumption probably somewhat overstates the impact of treatment and prophylaxis, because in reality individuals whose index cases have been treated may have greater access to prophylaxis due to better health care access and knowledge of their exposure to an infected index case. As a result, prophylaxed contacts may be on average at less risk of transmission than unprophylaxed contacts, since their index cases will be less infectious.

In this section we explore an alternate model structure in which a correlation (in principle negative or positive) is allowed between treatment of an index case and prophylaxis of his or her contacts. Here we assume that a fraction f_T of cases will be treated, and that a fraction f_{TP} of their contacts will be prophylaxed; on the other hand, a (probably lower) fraction f_{UP} of contacts of untreated cases will be prophylaxed. The total fraction of contacts prophylaxed (ignoring the fact that an individual may be a contact of multiple index cases) is then $f_T f_{TP} + (1 - f_T) f_{UP}$, and the model in the main text may be recovered by setting $f_{TP} = f_{UP} = f_P$. Complete correlation between treatment and prophylaxis, in which only contacts of treated index cases receive prophylaxis, comes from setting $f_{TP} = 1; f_{UP} = 0$. Note that in either case the total fraction prophylaxed is f_P.

The corresponding model equations would be:

$$\frac{dX}{dt} = -\lambda_{SU}(1 - f_{UP}e_P + f_{UP}e_P a_P)X - \lambda_{ST}(1 - f_{TP}e_P + f_{TP}e_P a_P)X - \lambda_R X$$

$$\frac{dY_{SU}}{dt} = \lambda_{SU}(1 - f_{UP})(1 - f_T)X + \lambda_{ST}(1 - f_{TP})(1 - f_T)X - vY_{SU}$$

$$\frac{dY_{ST}}{dt} = \lambda_{SU}[f_{UP}(1 - e_P - c_P) + (1 - f_{UP}) f_T (1 - c_T)]X + \lambda_{ST}[f_{TP}(1 - e_P - c_P) + (1 - f_{TP}) f_T (1 - c_T)]X - v_T Y_{ST}$$

$$\frac{dY_R}{dt} = f_{UP}c_P\lambda_{SU}X + (1 - f_{UP}) f_T c_T \lambda_{SU}X + f_{TP}c_P\lambda_{ST}X + (1 - f_{TP}) f_T c_T \lambda_{ST}X + \lambda_R X - v_R Y_R$$

$$\frac{dZ}{dt} = \lambda_{SU} f_{UP}e_P a_P X + \lambda_{ST} f_{TP}e_P a_P X + vY_{SU} + v_T Y_{ST} + v_R Y_R$$

$$\lambda_{ST} \equiv \beta_{ST}Y_{ST}$$

$$\lambda_{SU} \equiv \beta_{SU}Y_{SU}$$

$$\lambda_R \equiv \beta_R Y_R$$

Numerical solution of this model (Figure S1) confirms that in the extreme case in which treatment only applies to contacts of prophylaxed index cases, the effect is to reduce the net overall rate of antiviral use (with treatment not going to those in most need, namely those exposed to an unprophylaxed index case). The shapes of the curves for total infections and mean incidence time are very similar to those in the main text Figure 4 in the main text, except that the scale has been increased by 50%, now considering antiviral use in between 0 and 60% of cases/contacts, rather than up to 40% as in the main text.

6. Assessment of the impact of the quasi-stochastic approach to appearance of resistance.

We have used a deterministic model for this study, and have incorporated the stochastic, possibly very rare, event of emergence of a transmissible resistant strain during treatment or prophylaxis according to a scheme described in METHODS, where transmission of resistance from an age class is allowed to begin after the expected number of resistant infections emerging de novo during treatment or prophylaxis in this age group, combined with the expected number of infections transmitted from other age groups exceeds one. In reality, of course, these dynamics are stochastic, and resistance may appear before its expected appearance time, or may appear but stochastically go extinct in its first or first several appearances. To assess the impact of such stochastic variation, we varied the threshold for beginning transmission from an age group from 1/8 expected infections (Figure S2), to 1 (Figure 4, main text), to 8 (Figure S3).

As the figures show, there is little qualitative difference across these varying thresholds. Quantitatively, as one would expect, antiviral use is more effective, because resistance spreads later and less widely, as the threshold is increased. Indeed, setting the threshold *x* times higher is similar (though not quite identical) to setting the *de novo* resistance rates c_p and c_T in the model *x* times lower. This fact accounts for the strong resemblance between rows A-C in Figure S2 and rows D-F in Figure S3; the latter has a 100-fold higher *de novo* resistance probability and a 64-fold lower threshold.

7. Code for the numerical solutions. This code can be cut and pasted into Berkeley Madonna, a differential equation solver available in a free test version from www.berkeleymadonna.com

```
{Model for Lipsitch et al. PLoS Medicine}
{ANTIVIRAL RESISTANCE AND THE CONTROL OF PANDEMIC INFLUENZA}
METHOD euler

STARTTIME = 0
STOPTIME=500
DT = 0.1



{PART 1: BASIC EQUATIONS}
{PART 1A: IN TERMS OF FORCES OF INFECTION (lambda)}
d/dt(X[1..6]) = u - lambdaS[i] * (1-fp*ep)*X[i] - lambdaR[i]*X[i]- u*X[i] -lambdaS[i]*X[i]*fp*ep*ap
d/dt(YSu[1..6]) = (1-fp)*(1-ft)*lambdaS[i]*X[i] - v*YSu[i] - u*YSu[i]
d/dt(YSt[1..6]) = lambdaS[i]*X[i]*(fp*(1-ep-cp)+(1-fp)*ft*(1-ct)) - vt*YSt[i] - u*YSt[i]
d/dt(YR[1..6]) =(lambdaS[i]*fp*cp*X[i] + ft*lambdaS[i]*(1-fp)*X[i]*ct +lambdaR[i]*X[i] - vr*YR[i] -
u*YR[i])*whirlwind[i]
d/dt (Z[1..6]) = lambdaS[i]*fp*ep*ap*X[i]+ v*YSu[i]+vt*YSt[i]+vr*YR[i] - u*Z[i]

{we use "whirlwind" for the Greek letter xi to avoid confusion with X and i}


{PART 1B: THE FOI THEMSELVES}
lamS1[1..6]=bSU[1,i]*ySu[i]+bST[1,i]*YSt[i]
lambdaS[1]=arraysum(lamS1[*])

lamS2[1..6]=bSU[2,i]*ySu[i]+bST[2,i]*YSt[i]
lambdaS[2]=arraysum(lamS2[*])

lamS3[1..6]=bSU[3,i]*ySu[i]+bST[3,i]*YSt[i]
lambdaS[3]=arraysum(lamS3[*])

lamS4[1..6]=bSU[4,i]*ySu[i]+bST[4,i]*YSt[i]
lambdaS[4]=arraysum(lamS4[*])

lamS5[1..6]=bSU[5,i]*ySu[i]+bST[5,i]*YSt[i]
lambdaS[5]=arraysum(lamS5[*])

lamS6[1..6]=bSU[6,i]*ySu[i]+bST[6,i]*YSt[i]
lambdaS[6]=arraysum(lamS6[*])

{NOTE: HERE WE USE yRt, not yR, to allow for whirlwind}
lamR1[1..6] = bR[1,i]*yRt[i]
lambdaR[1]= arraysum(lamR1[*])

lamR2[1..6] = bR[2,i]*yRt[i]
lambdaR[2]= arraysum(lamR2[*])

lamR3[1..6] = bR[3,i]*yRt[i]
lambdaR[3]= arraysum(lamR3[*])
```

```
lamR4[1..6] = bR[4,i]*yRt[i]
lambdaR[4]= arraysum(lamR4[*])

lamR5[1..6] = bR[5,i]*yRt[i]
lambdaR[5]= arraysum(lamR5[*])

lamR6[1..6] = bR[6,i]*yRt[i]
lambdaR[6]= arraysum(lamR6[*])


{PART 2: KEEP TRACK OF AUXILIARY QUANTITIES OF INTEREST}

{PART 2A: SUMMING ACROSS TREATMENT GROUPS AND AGE GROUPS TO GET TOTALS
AND PROPORTIONS, PLUS TOTAL POPULATION SIZE N}

YS[1..6]=YSu[i]+YSt[i]
SUMYS=arraysum(YS[*])
SUMYR=arraysum(YR[*])
SUMX=arraysum(X[*])

PROPYS=arraysum(YS[*])/N
PROPYR=arraysum(YR[*])/N
PROPX=arraysum(X[*])/N

N =  SUMYS+SUMYR+SUMX+arraysum(Z[*])+arraysum(Zpro[*])


{PART 2B: CALCLUATIONS FOR FIGURE 2E-G}

d/dt(cumIncS[1..6])=(1-fp)*(1-ft)*lambdaS[i]*X[i] +lambdaS[i]*X[i]*(fp*(1-ep-cp)+(1-fp)*ft*(1-ct))
d/dt(cumIncR[1..6])=lambdaS[i]*fp*cp*X[i] + ft*lambdaS[i]*(1-fp)*X[i]*ct +lambdaR[i]*X[i] {this is
also used for calculation of whirlwind, later}
SUMCumIncR=arraysum(cumIncR[*])
SUMCumIncS=arraysum(cumIncS[*])
PROPCumIncS=SUMCumIncS/N
PROPCumIncR=SUMCumIncR/N
PROPCumInc=PROPcumIncR+PROPcumIncS
G=IF SUMCumIncS+SUMCumIncR>0 THEN SUMcumincR/(SUMCumIncS+SUMCumIncR)
ELSE 0 {Cumulative fraction of cases resistant -- orange curves in Fig. 2E-G}


ResAcquired[1..6] = lambdaS[i]*fp*cp*X[i] + ft*lambdaS[i]*(1-fp)*X[i]*ct
ResTransmitted[1..6]=lambdaR[i]*X[i]
SUMResAcquired=arraysum(ResAcquired[*])
SUMResTransmitted=arraysum(ResTransmitted[*])
H=IF (SUMResTransmitted+SUMResAcquired >0 )THEN SUMResAcquired/
(SUMResTransmitted+SUMResAcquired) ELSE 1 {Instantaneous proportion of resistant
infections through acquired route -- black curves in Fig. 2E-G}


{PART 2C: CALCULATING WHIRLWIND AND THE VALUE OF YRt, which is actually used for
the forces of infection}
```

```
whirlwind[1..6]= switch(cumincR[i],1)  {We let the YR compartment stay at 1 (see the YR updater)
and not transmit (see below) until cumulative incidence reaches 1}
YRt[1..6]=whirlwind[i]*YR[i]  {the contribution of YRt to transmission is 0 until the compartment is
allowed to "take off" at which point it is also set to 1}


{PART 2D: CALCULATING MEAN TIME OF CASE INCIDENCE: DASHED CURVES IN FIG. 4}
d/dt(SUMStrataIncTime[1..6])=TIME*(lambdaS[i]*fp*cp*X[i] + ft*lambdaS[i]*(1-fp)*X[i]*ct
+lambdaR[i]*X[i] +(1-fp)*(1-ft)*lambdaS[i]*X[i] +lambdaS[i]*X[i]*(fp*(1-ep-cp)+(1-fp)*ft*(1-ct)))
SUMIncTime=Arraysum(SUMStrataIncTime[*])
MEANIncTime=IF SUMCumIncS+SUMCumIncR>0 THEN
SUMIncTime/(SUMCumIncS+SUMCumIncR) ELSE 0


{PART 2E: MISCELLANEOUS}

d/dt (Zpro[1..6]) = lambdaS[i]*X[i]*fp*ep*ap
PROPZPRO=arraysum(Zpro[*])/N {here we keep track of individuals who are exposed and
infected while on prophylaxis, becoming immune without being infectious}
ZProPerRecovered=PROPZPRO/(1-PROPX)

pRes = SUMYR/(SUMYS+SUMYR) {instantaneous proportion of cases that are resistant}

{PART 3: PARAMETER VALUES}
R0=1.8
ft = 0.3 {frequency of treatment}
fp = ft {0.3}{frequency of propylaxis}
cp=2e-4
ct=cp*10
fitcost =0.1
u = 0 {ignore birth and death}
bSU[1..6,1..6]=beta[i,j]
bSt[1..6,1..6]=bSu[i,j]*(1-ei)^(1-gamma)
bR[1..6,1..6] = bSu[i,j]*(1-fitcost)
v = 0.3 {3.33 day duration}
vt = v/(1-ei)^gamma {duration for treated}
vr=v {no duration fitness cost}
ep = .85 {efficacy of prophylaxis in preventing infection (aveS)}
ei = 0.66
gamma =0
ap = 0


{PART 4: INITIALIZATIONS}
init X[1..6]=pop[i]-1
init YSu[1..6] = 1
init YSt[1..6]=0
init YR [1..6]=1
init Z [1..6]= .0
init Zpro[1..6]=0

init cumIncR[1..6]=0
init cumIncS[1..6]=0
init SUMStrataIncTime[1..6]=0
```

```
{PART 5: DEMOGRAPHICS AND WAIFW MATRIX}

k[1,1]=169.14
k[1,2]=31.47
k[1,3]=17.76
k[1,4]=34.5
k[1,5]=15.83
k[1,6]=11.47
k[1..6,1]=k[1,i]
k[2,2]=274.51
k[2,3]=32.31
k[2,4]=34.86
k[2,5]=20.61
k[2,6]=11.5
k[2..6,2]=k[2,i]
k[3,3]=224.25
k[3,4]=50.75
k[3,5]=37.52
k[3,6]=14.96
k[3..6,3]=k[3,i]
k[4,4]=75.66
k[4,5]=49.45
k[4,6]=25.08
k[4..6,4]=k[4,i]
k[5,5]=61.26
k[5,6]=32.99
k[5..6,5]=k[5,i]
k[6,6]=54.23
{k[1..6,1..6]=1}


pop[1]=960000
pop[2]=1265000
pop[3]=1642000
pop[4]=4857000
pop[5]=3312000
pop[6]=2477000
{pop[1..6]=1.45e7/6}
poptot=arraysum(pop[*])

beta[1..6,1..6]=R0/47.35*k[i,j]/poptot*(v+u)
```

REFERENCES

1. Wallinga J, Kretzschmar M, Teunis P (2006) Using social contact data to estimate age-specific transmission parameters for infectious respiratory spread agents. Am J Epidemiol.
2. Anderson RM, May RM (1991) Infectious Diseases of Humans: Dynamics and Control. Oxford: Oxford University Press.
3. Diekmann O, Heesterbeek JAP (2000) Mathematical Epidemiology of Infectious Diseases: Model Building, Analysis and Interpretation. New York: John Wiley & Sons.

Fig. S1: Structural sensitivity analysis for correlated prophylaxis and treatment. This figure recapitulates Figure 4 in the main text – which shows the effect of varying effective reproductive numbers (R_E) and of antiviral use on total attack rate (solid curves) and mean incidence time (dashed curves) – under the assumption that treatment is offered only to contacts of prophylaxed hosts. This is an extreme assumption (the opposite extreme to the uncorrelated use of treatment and prophylaxis assumed in the main text) designed to explore the sensitivity of the model to this assumption. Qualitative results are nearly identical to those shown in Figure 4, but the effect of antivirals is scaled down by roughly 1/3 for the parameters used here; note that the figures are nearly identical but the horizontal scale here goes up to 60% antiviral use, rather than 40% in Fig. 4.

Fig. S2: Sensitivity analysis for the threshold for takeoff of the resistant strain: low threshold. This figure recapitulates Figure 4 in the main text under the assumption that resistant strains can spread from a given age group when the expected number of resistant infections has reached 1/8 (rather than 1 in Figure 4). The no-resistance case is not shown since this would be identical to Figure 4.

Fig. S3: Sensitivity analysis for the threshold for takeoff of the resistant strain: high threshold. This figure recapitulates Figure 4 in the main text under the assumption that resistant strains can spread from a given age group when the expected number of resistant infections has reached 8 (rather than 1 in Figure 4). The no-resistance case is not shown since this would be identical to Figure 4.

FIGURE S1

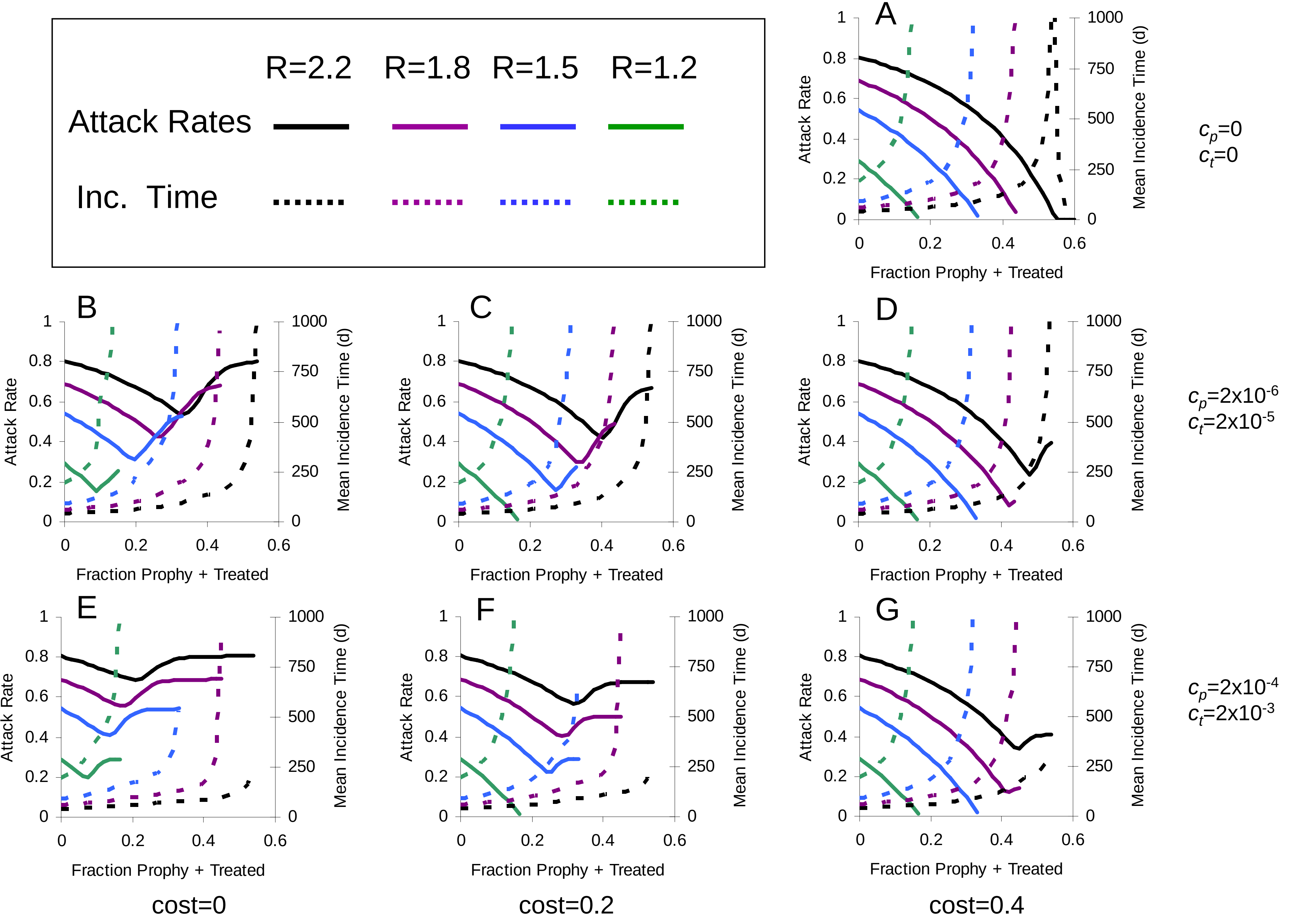

R=2.2
R=1.8
R=1.5
R=1.2
Attack Rates
Inc. Time
A
B
C
D
E
F
G
Attack Rate
Mean Incidence Time (d)
Fraction Prophy + Treated
c_p=0
c_t=0
c_p=2x10^{-6}
c_t=2x10^{-5}
c_p=2x10^{-4}
c_t=2x10^{-3}
cost=0
cost=0.2
cost=0.4

FIGURE S2

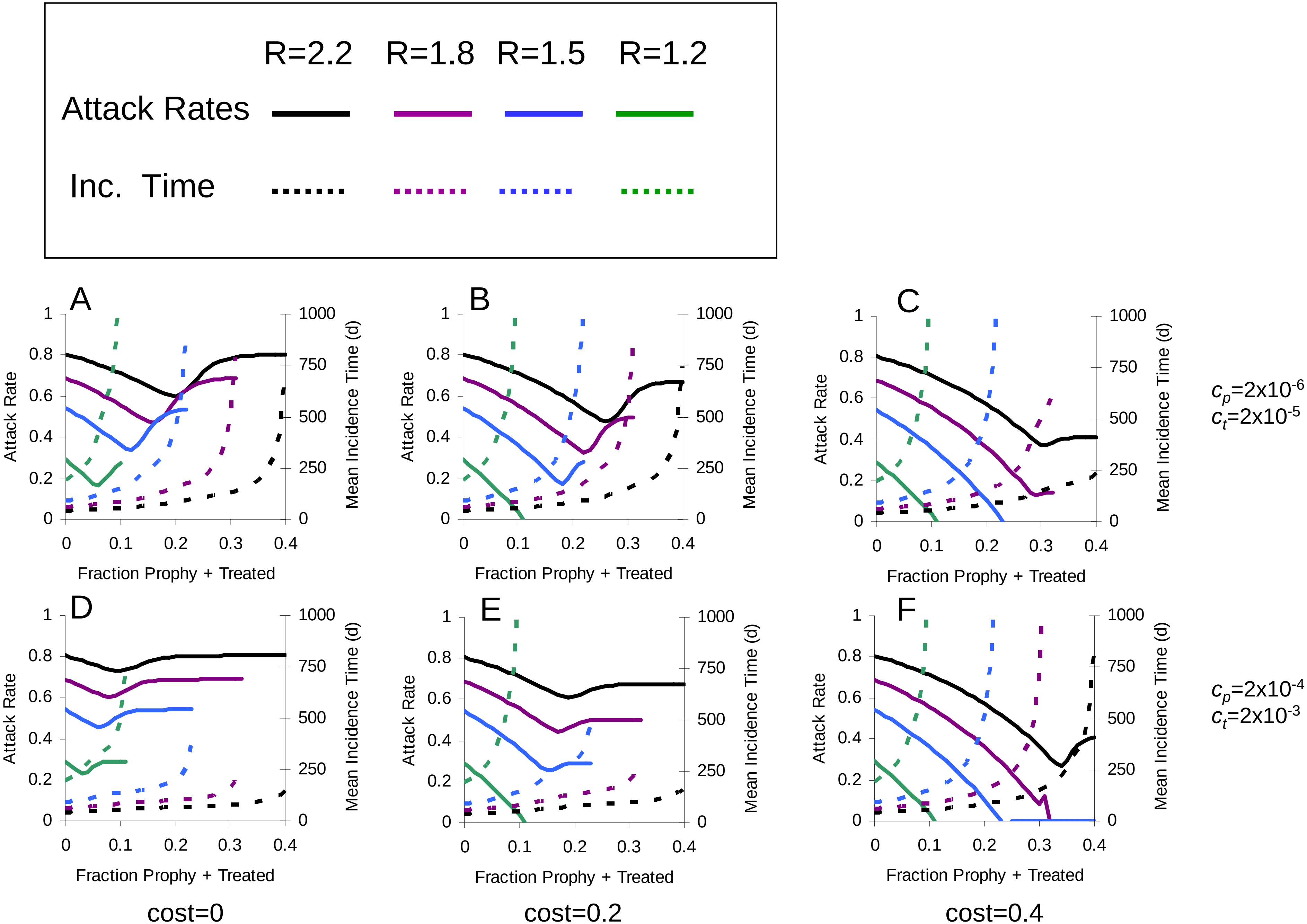
R=2.2
R=1.8
R=1.5
R=1.2
Attack Rates
Inc. Time
A
B
C
D
E
F
Attack Rate
Mean Incidence Time (d)
Fraction Prophy + Treated
0
0.1
0.2
0.3
0.4
0.6
0.8
1
250
500
750
1000
c_p=2x10^{-6}
c_t=2x10^{-5}
c_p=2x10^{-4}
c_t=2x10^{-3}
cost=0
cost=0.2
cost=0.4

FIGURE S3

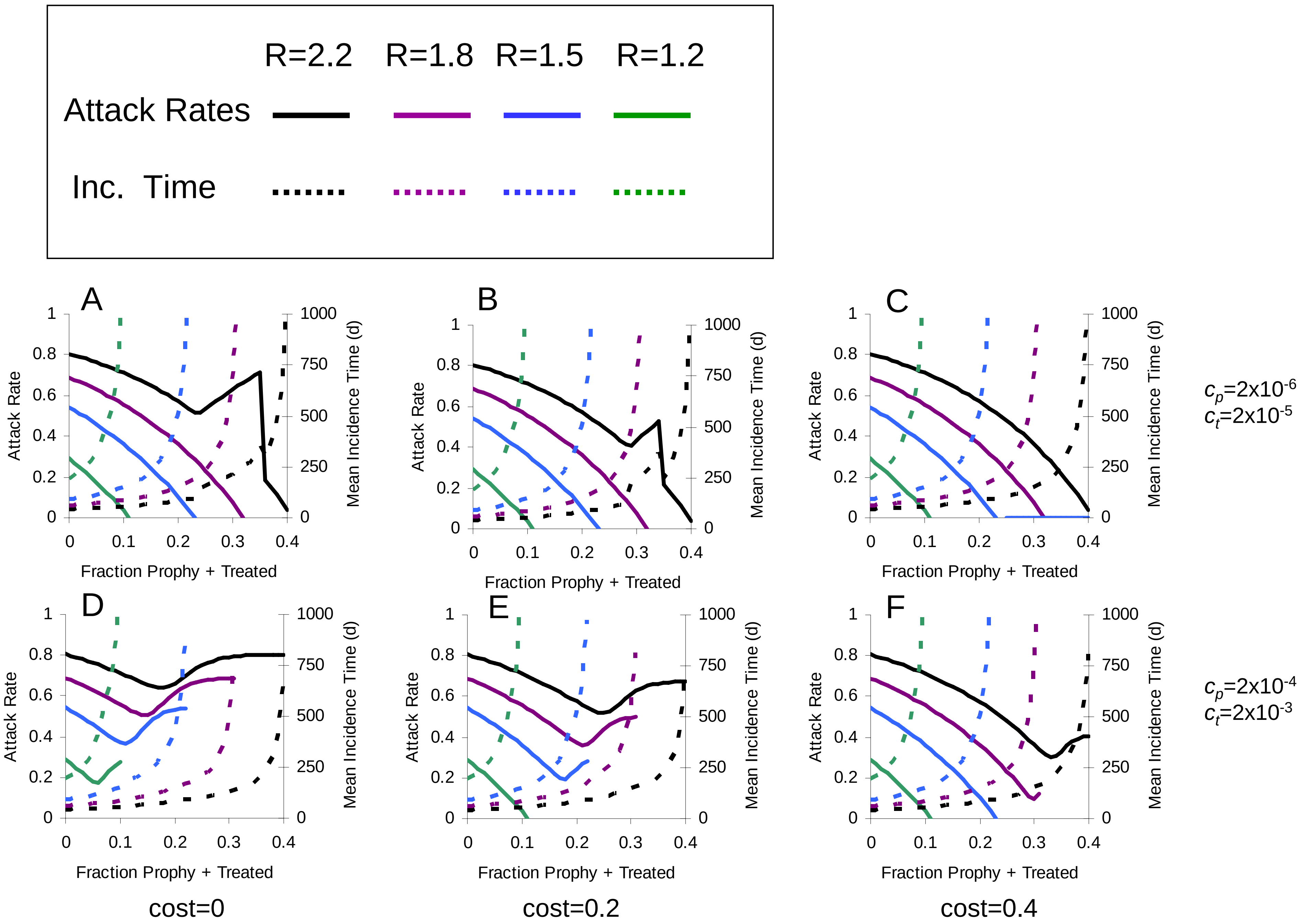
R=2.2
R=1.8
R=1.5
R=1.2
Attack Rates
Inc. Time
A
B
C
D
E
F
Attack Rate
Mean Incidence Time (d)
Fraction Prophy + Treated
0
0.1
0.2
0.3
0.4
0.6
0.8
1
250
500
750
1000
c_p=2x10^{-6}
c_t=2x10^{-5}
c_p=2x10^{-4}
c_t=2x10^{-3}
cost=0
cost=0.2
cost=0.4
