# Supplementary material for: Antiviral Resistance and the Control of Pandemic Influenza
Source: PLoS Med. 2007 Jan 23;4(1):e15. doi: 10.1371/journal.pmed.0040015 (PMC1779817; doi:10.1371/journal.pmed.0040015)
Supplement: Supplementary file 2 [file pmed.0040015.st001.doc]

TABLE S1:  Population sizes and transmission coefficients (WAIFW matrix [2]) for the age-structured model, adapted from [1].  The daily transmission rate constant in row i, column j, indicates that an infected individual in age group j has a daily probability of transmitting infection  to each of the susceptible individuals in group i.   Note that table entries are inflated by a factor of 10^9^ for readability.  This matrix is calibrated for .  

	Daily transmission rate constant  / 10^9^
	
Population Size
(millions)	Age group (y)	0-5	6-12	13-19	20-39	40-59	60+	
0.96	0-5	98.5	18.3	10.3	20.1	9.2	6.7	
1.27	6-12	18.3	159.8	18.8	20.3	12.0	6.7	
1.64	13-19	10.3	18.8	130.5	29.5	21.8	8.7	
4.86	20-39	20.1	20.3	29.5	44.0	28.8	14.6	
3.31	40-59	9.2	12.0	21.8	28.8	35.7	19.2	
2.48	60+	6.7	6.7	8.7	14.6	19.2	31.6	

References

1. Wallinga J, Kretschmar M, Teunis P (2006) Using social contact data to estimate age-specific transmission parameters for infectious respiratory spread agents. Am J Epidemiol

2. Anderson RM, May RM (1991) Infectious Diseases of Humans: Dynamics and  Control. Oxford: Oxford University Press.
